# Investigation of the mechanism of Xiaoyin Jiedu Yin in the treatment of psoriasis based on bioinformatics, machine learning

**DOI:** 10.3389/fchem.2025.1623449

**Published:** 2025-07-15

**Authors:** Ru-Nan Fang, Yang Zhou, Yang Shen, Yuan Sun, Jian-Hong Li

**Affiliations:** 1 Department of Dermatology, Dongzhimen Hospital, Beijing University of Chinese Medicine, Beijing, China; 2 Department of Endocrinology, Guang’anmen Hospital, Beijing University of Chinese Medicine, Beijing, China

**Keywords:** psoriasis, bioinformatics, network pharmacology, Xiaoyin Jiedu Yin, machine learning, single-cell RNA sequencing

## Abstract

**Introduction:**

Psoriasis is a chronic immune-mediated inflammatory skin disease. Xiaoyin Jiedu Decoction (XYJDY) is a traditional Chinese medicinal formula that has demonstrated significant clinical efficacy in alleviating psoriatic symptoms; however, its underlying pharmacological mechanisms remain unclear.

**Methods:**

We employed network pharmacology, machine learning–based target screening, and functional enrichment to identify key targets and pathways. Single-cell RNA sequencing (scRNA-seq) and spatial transcriptomics (ST) were used to validate gene expression. An IL-17A–induced HaCaT cell model was established for *in vitro* validation.

**Results:**

AKR1B10 was identified as the core therapeutic target of XYJDY in psoriasis. It was markedly upregulated in psoriatic skin lesions, primarily enriched in keratinocytes, and its expression demonstrated positive correlations with multiple pro-inflammatory immune cell subsets. *In vitro* experiments showed that XYJDY-medicated serum significantly downregulated AKR1B10 expression in IL–17A–stimulated HaCaT cells.

**Conclusion:**

This study reveals that the multi-component formula XYJDY exerts anti-psoriatic effects through a multi-target synergistic mechanism, in which AKR1B10 is a potential core target. These findings provide a theoretical foundation for further exploration of the molecular mechanisms underlying the efficacy of XYJDY in psoriasis treatment.

## 1 Introduction

Psoriasis is a chronic, immune-mediated inflammatory skin disease marked by genetic predisposition and autoimmune dysregulation. It affects approximately 125 million people globally, with 80%–90% of cases presenting as plaque-type psoriasis (Armstrong and Read, 2020). Beyond its cutaneous manifestations, psoriasis is strongly linked to a range of systemic comorbidities, including inflammatory arthritis, cardiometabolic disorders, mental health conditions, and gastrointestinal diseases (Takeshita et al., 2017).

Traditional Chinese Medicine (TCM), as a vital component of complementary and alternative medicine, has shown promising adjunctive effects in the treatment of various chronic conditions (L. Zhao et al., 2023). From the TCM perspective, the core pathogenesis of psoriasis is attributed to heat-toxicity in Xuefen. Xiaoyin Jiedu Yin (XYJDY), a classical herbal formula, consists of Buffalo Horn, Japanese Honeysuckle Flower Bud, Indigowoad Root, Bistort Rhizome, Tree Peony Root–bark, Adhesive Rehmannia Root Tuber, Red Paeoniae Trichocarpae, Redroot Gromwell, Chinese Manyleaf Paris Rhizome, Lightyellow Sophra Root, Densefruit Pittany Root-bark, Glabrous Greenbrier Rhizome, and Liquorice Root. The formulation is traditionally used to clear heat and eliminate toxins, cool the blood, and resolve blood stasis. Its therapeutic efficacy has been consistently validated through over 30 years of clinical application.

The rapid advancements in bioinformatics and network pharmacology have revolutionized research paradigms in TCM, offering novel insights into complex disease mechanisms. In this study, we developed a comprehensive, multidimensional integrative analysis framework to systematically elucidate the molecular mechanisms underlying the therapeutic effects of XYJDY in psoriasis. We first curated psoriasis-related genetic targets from multiple reputable bioinformatics databases. Active compounds within XYJDY were identified through network pharmacology techniques, and shared targets between the decoction and psoriasis were subjected to functional annotation and pathway enrichment analyses. Machine learning algorithms were applied to rank potential therapeutic targets to refine our findings, ultimately highlighting aldo-keto reductase family 1 member B10 (AKR1B10) as a pivotal molecular target.

Building upon these insights, we conducted a comprehensive analysis of immune microenvironmental infiltration, integrating single-cell RNA sequencing (scRNA-seq) and spatial transcriptomics (ST) technologies to validate the expression patterns and spatial localization of AKR1B10 in psoriasis-associated tissues across multiple biological layers. Furthermore, an IL-17A-induced psoriasiform inflammation model in HaCaT keratinocytes demonstrated that XYJDY markedly suppressed AKR1B10 expression, reinforcing its candidacy as a pivotal therapeutic target in XYJDY-mediated treatment of psoriasis.

In summary, this study employed an integrative technological approach to delineate the key targets, and underlying mechanisms of XYJDY in psoriasis therapy, thereby providing compelling evidence for the multi-target regulatory paradigm of traditional Chinese medicine in complex disease management.

## 2 Materials and methods

### 2.1 Active components and targets of XYJDY

Thirteen herbal constituents of XYJDY were systematically analyzed using the Traditional Chinese Medicines Network Pharmacology Analysis System (TCMNPAS), an integrated platform encompassing five widely utilized databases: HIT (Herbal Ingredient’s Targets), TCMID (Traditional Chinese Medicines Integrated Database), TCMSP (Traditional Chinese Medicines Systems Pharmacology Database), STITCH, and a custom-built database (Liu et al., 2024). Active compounds and their associated targets were identified based on the following screening parameters: a quantitative estimate of drug-likeness (QED) ≥ 0.2, a drug–target association score ≥400, and a compound–target significance threshold of *P* < 0.05.

### 2.2 Psoriasis-related disease targets

Microarray datasets associated with psoriasis were retrieved from the GEO public database (https://www.ncbi.nlm.nih.gov/geo/), with non-human gene chips excluded. Datasets GSE78097, GSE182740, and GSE226244 were selected as the training cohort, and GSE201827 was used as an independent validation set. Gene annotation was performed using Perl, converting probe-level data into gene-level expression matrices based on the corresponding platform annotation files. The combined training data were normalized and analyzed for differential gene expression using the “limma” package in R (version 4.0.2). Differentially expressed genes (DEGs) were identified with thresholds set at *P* < 0.05 and |logFC| > 0.5.

To complement this analysis, psoriasis-related genes were retrieved by searching the keyword “psoriasis” in the GeneCards, OMIM, and DisGeNET databases. The intersection of DEGs and psoriasis-related genes was visualized using Venny 2.1.0, resulting in the identification of candidate psoriasis-specific disease targets.

### 2.3 Intersection genes of XYJDY and psoriasis and construction of the XYJDY drug–active ingredient–target network

The targets of XYJDY were overlapped with psoriasis-associated targets, and a Venn diagram was constructed in Venny 2.1.0 to visualize and pinpoint the shared targets through which XYJDY may exert therapeutic effects in psoriasis. Subsequently, data on XYJDY’s constituent herbs, their bioactive compounds, and corresponding targets were imported into Cytoscape 3.9.1 to assemble the XYJDY drug–active ingredient–target network. A topological analysis of this network was then performed to pinpoint the key bioactive compounds responsible for XYJDY’s therapeutic effects on psoriasis.

### 2.4 GO and KEGG enrichment analyses

Gene Ontology (GO) and Kyoto Encyclopedia of Genes and Genomes (KEGG) pathway enrichment analyses were performed on the intersecting target genes using the “clusterProfiler” package in R. Enrichment results with a P-value <0.05 were deemed statistically significant.

### 2.5 Machine learning-based identification of key targets and external dataset validation

Three machine-learning algorithms were applied to the intersecting gene set to identify key therapeutic targets. The least absolute shrinkage and selection operator (LASSO) is a linear regression technique incorporating L1 regularization, simultaneously performing variable selection and regularization (Kang et al., 2021). By shrinking regression coefficients, LASSO achieves dimensionality reduction and model optimization. The support vector machine–recursive feature elimination (SVM-RFE) algorithm ranks genes based on their importance using a support vector machine and recursively eliminates features to identify the most informative subset—defined as the set with the lowest classification error (Li et al., 2024). These are considered the SVM-RFE-derived feature genes. The random forest (RF) algorithm, renowned for its robustness in analyzing high-dimensional data, integrates multiple decision trees to enhance predictive accuracy and model stability (X. Zhao et al., 2025). The gene sets selected by these three machine learning approaches are not only valuable for constructing high-performance classification or predictive models but also offer important insights into the molecular mechanisms of disease and the identification of therapeutic targets. As such, they represent powerful tools for biomarker discovery and functional genomics research. Therefore, this study performed an intersection analysis of the feature genes predicted by the three models, and the overlapping genes were regarded as the key targets of XYJDY in the treatment of psoriasis.

The validation dataset GSE201827 was used to verify the differential expression of the key targets between the normal and psoriasis groups. Receiver Operating Characteristic (ROC) curves were plotted for the key target genes, and those with an Area Under the Curve (AUC) greater than 0.7 and *P* < 0.05 were selected as the final key targets.

### 2.6 CIBERSORT and immune infiltration analysis

In this study, we utilized the CIBERSORT algorithm to estimate the relative abundance of immune cell subsets across three psoriasis-related training datasets. Spearman correlation analysis was performed to evaluate the associations between the therapeutic target genes of XYJDY and the infiltration levels of specific immune cell types. Correlations with a *P*-value <0.05 were deemed statistically significant. Data visualization was carried out using the “ggplot2” package in R.

### 2.7 Processing of scRNA-seq data

The scRNA-seq datasets analyzed in this study were retrieved from the GEO database (accession numbers: GSE220116 and GSE230842), each comprising both healthy and psoriatic skin samples. Data processing and merging were performed using R software (version 1.4.2) in conjunction with the “Seurat” package, applying the merge function to integrate datasets. Genes with low expression levels were filtered out, and the resulting dataset was normalized. Principal component analysis (PCA) and uniform manifold approximation and projection (UMAP) were then conducted for dimensionality reduction and visualization. Cell type annotation was performed using the “SingleR” package, and DEGs across cell clusters were identified using the FindAllMarkers function. Final cell type identities were determined based on canonical marker genes.

### 2.8 Processing and analysis of ST data

Spatial transcriptomics data (GSE251950) were obtained from the GEO database. In R (version 1.4.2), raw gene expression matrices were imported using the Read10X function, and spatial coordinate metadata were integrated using Read10X_Image. Separate “Seurat” objects were constructed for each individual sample. To minimize technical variability and ensure consistency across samples, normalization was performed using the SCTransform algorithm, resulting in a harmonized dataset for downstream analysis. Dimensionality reduction was achieved through PCA, followed by cell clustering based on UMAP and the shared nearest neighbor (SNN) modularity optimization algorithm. DEGs between clusters were identified using the FindAllMarkers function with thresholds of |log2FC| > 1 and adjusted *P* < 0.05. Group-level expression differences between psoriasis and control samples were quantified using pseudobulk analysis. The spatial expression patterns of key genes were visualized with the SpatialFeaturePlot function.

### 2.9 Experiments

#### 2.9.1 Animals

Twenty male specific pathogen-free (SPF) Sprague-Dawley (SD) rats (six to eight weeks old) were obtained from Speifu (Beijing) Biotechnology Co., Ltd. (Beijing, China, Approval No. SYXK (Beijing) 2021-0017). The animals were maintained under standard laboratory conditions, including a controlled temperature of 22°C–26°C, relative humidity of 40%–70%, and a 12-h light/dark cycle. Rats were provided with unrestricted access to food and water. All animals were acclimatized for 1 week prior to oral gavage and were used to prepare XYJDY-containing serum. Animal welfare and experimental procedures were carried out in strict accordance with the guidelines approved by the Animal Ethics Committee of the China Academy of Chinese Medical Sciences (Ethical Approval No. ERCCACMS21-2410-02).

#### 2.9.2 Traditional Chinese medicine

The Chinese herbal formula XYJDY, administered at a dosage of 34 g per packet twice daily, comprises 13 medicinal herbs, as detailed in Table 1. The formulation was provided by the granule pharmacy of Dongzhimen Hospital, Beijing University of Chinese Medicine, and was manufactured by Beijing Kangrentang Pharmaceutical Co., Ltd.

**TABLE 1:** Information for XYJDY granule composition.

Chinese name	Herb’s English name	Herb’s Latin name
Shui Niu Jiao (SNJ)	Buffalo Horn	Bubali Cornu
Jing Yin Hua (JYH)	Japanese Honeysuckle Flower Bud	Lonicerae Japonicae Flos
Ban Lan Gen (BLG)	Isatidis Radix	Indigowoad Root
QUAN SHEN (QS)	Rhizome of Bistort	Rhizoma Bistortae
Mu Dan Pi (MDP)	Tree Peony Root - bark	Moutan Cortex
Sheng Di Huang (SDH)	Adhesive Rehmannia Root Tuber	Rehmanniae Radix
Chi Shao (CS)	Red Paeoniae Trichocarpae	Paeoniae Radix Rubra
Zi Cao (ZC)	Redroot Gromwell	Arnebiae Radix
Chong Lou (CL)	Chinese Manyleaf Paris Rhizome	Paridis Rhizoma
Ku Shen (KS)	Lightyellow Sophra Root	Sophorae Flavescentis Radix
Bai Xian Pi (BXP)	Denesefruit Pittany Root-bark	Dictamni Cortex
Tu Fu Lin (TFL)	Glabrous Greenbrier Rhizome	Smilacis Glabrae Rhizoma
GAN CAO (GC)	Liquorice Root	Glycyrrhizae Radix et Rhizoma

#### 2.9.3 Preparation of serum containing the XYJDY decoction

After a 1-week acclimatization period, twenty male Sprague-Dawley rats were randomly divided into two groups (n = 10 per group): a control group and a drug-containing serum group. Rats in the control group received 0.9% saline by oral gavage (10 mL kg^-1^), while those in the drug-containing group were administered an XYJDY suspension at a dosage of 3 g kg^-1^, determined based on the standard human-to-rat body surface area conversion. Gavage was performed once daily for 7 consecutive days. One hour following the final administration, the animals were anesthetized with isoflurane, and blood samples were collected from the abdominal aorta. Samples were centrifuged at 3,000 rpm for 15 min (centrifugal radius: 10 cm), and the resulting supernatants were incubated at 56°C for 30 min. The serum was then sterilized by filtration through a 0.2 μm microporous membrane. The final preparations of normal rat serum and XYJDY-containing serum were aliquoted and stored at −80 °C for subsequent experiments. The serum obtained from the SD rats was used exclusively for *in vitro* cell experiments.

#### 2.9.4 Cell culture, treatment, and experimental grouping

Human immortalized keratinocytes (HaCaT cells; Fuheng Biological) were cultured in Dulbecco’s Modified Eagle Medium (DMEM) supplemented with 10% fetal bovine serum (FBS) and 1% penicillin-streptomycin (P/S) and maintained in a humidified incubator at 37 °C with 5% CO_2_ until reaching the logarithmic growth phase. To simulate the pathological changes of psoriasis, we adopted the IL-17A-stimulated HaCaT cell model, which has been widely used in psoriasis research to assess the effects of inflammatory factors on keratinocyte proliferation and gene expression (Shen et al., 2025). Proliferating HaCaT cells were seeded into culture plates and treated with varying concentrations of interleukin-17A (IL-17A; 50, 100, and 200 ng/mL; ILA-H82Q1, ACROBiosystems). Cell viability was assessed using the Cell Counting Kit-8 (CCK-8) assay at 24 and 48 h post-treatment. The concentration and time point that yielded the greatest enhancement in cell viability were defined as the optimal stimulation conditions.

Cells stimulated with the optimal IL-17A concentration were then treated with either blank control serum or XYJDY-medicated serum at concentrations of 5%, 10%, 15%, and 20%. CCK-8 assays were conducted at 0, 24, 48, and 72 h to evaluate cell viability across groups. The optimal medicated serum concentration and exposure duration were determined based on proliferation rates and applied in subsequent experiments.

Finally, IL-17A-induced HaCaT cells were assigned to two treatment groups: one group was given XYJDY-containing serum for intervention, one group was given blank control serum as a control group, and the other set of untreated HaCaT cells was a blank control group.

#### 2.9.5 Western blotting

HaCaT cells from the experimental groups described in Section 2.9.4 were collected, and total protein was extracted using RIPA lysis buffer supplemented with phenylmethylsulfonyl fluoride (PMSF) and phosphatase inhibitors. Proteins were separated by SDS-PAGE and transferred onto polyvinylidene difluoride (PVDF) membranes, followed by blocking with an appropriate blocking buffer. The membranes were incubated overnight at 4°C with a primary antibody against AKR1B10 (YT6598, ImmunoWay). After thorough washing with phosphate-buffered saline (PBS), the membranes were incubated at 27°C for 1 h with horseradish peroxidase (HRP)-conjugated goat anti-rabbit IgG (H + L) secondary antibody (S004, Tiandeyue). Beta-tubulin (YM3030, ImmunoWay) was used as an internal loading control. Protein bands were visualized using an enhanced chemiluminescence (ECL) detection system, and band intensities were quantified using ImageJ software.

#### 2.9.6 Real-time quantitative PCR (RT-qPCR) analysis of gene expression

Total RNA was extracted from the HaCaT cells in each experimental group (as described in Section 2.9.4) using TRNzol Total RNA Extraction Reagent (DP424, Tiangen) following phosphate-buffered saline (PBS) washing. First-strand cDNA was synthesized using the PrimeScript™ RT reagent kit with gDNA Eraser (Takara) according to the manufacturer’s instructions. Quantitative real-time PCR (qRT-PCR) was carried out on a QuantStudio™ 5 Real-Time PCR System (Applied Biosystems) using gene-specific primers and BlazeTaq™ SYBR^®^ Green qPCR Mix 2.0 (QP031-S, GeneCopoeia). Relative mRNA expression levels were calculated using the comparative Cq method (2^–ΔΔCq)^. β-actin was used as the internal reference gene. The primer sequences were as follows:

AKR1B10-F: CCC​AGG​AGA​CAG​AGG​TTA​TA;

AKR1B10-R: GAA​ATG​ATT​CTG​AGT​GAG​CAG​GTA​G.

β-Actin-F: TCC​TCC​TGA​GCG​CAA​GTA​CTC​C;

β-Actin-R: CAT​ACT​CCT​GCT​TGC​TGA​TCC​AC.

#### 2.9.7 Statistical analysis

Statistical analyses were performed using GraphPad Prism (v10.1.2). Data normality was assessed with the Kolmogorov-Smirnov test. For two-group comparisons, the Mann-Whitney test (non-parametric) or Student’s t-test (parametric) was used. One-way ANOVA followed by a Bonferroni *post hoc* test was applied for comparisons among multiple groups. Statistical significance was defined as *P* < 0.05.

## 3 Results

### 3.1 Network pharmacology

#### 3.1.1 Identification of potential compounds and targets of XYJDY related to psoriasis

**FIGURE 1:**
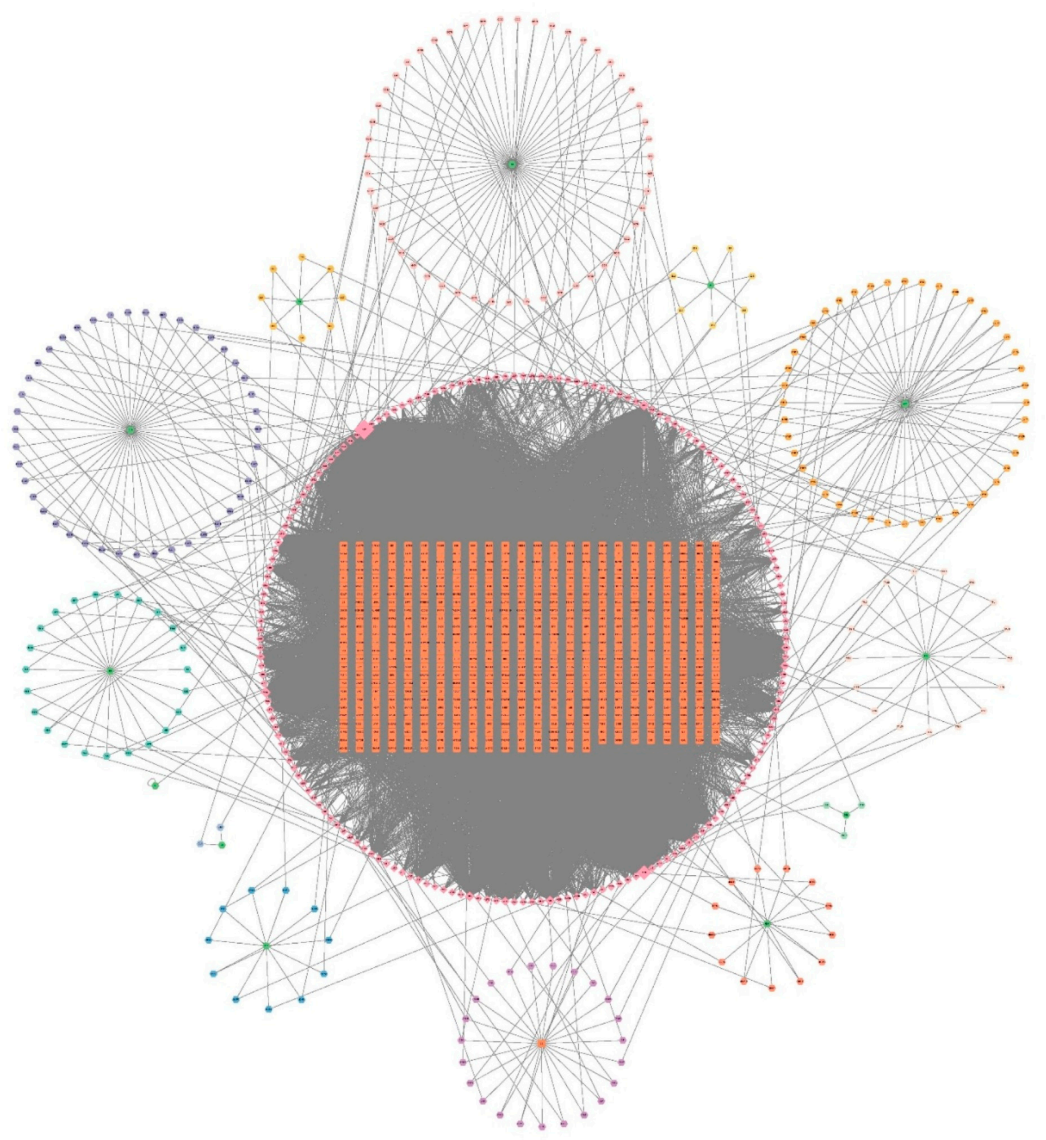
Drug-Active Ingredient Target Network for XYJDY. The green color represents the herbs, the circle around each herb represents its chemical composition, the orange color represents all the gene targets of XYJDY, and the pink color in the outer circle represents all the chemical compounds of XYJDY. Abbreviations: SNJ, Buffalo Horn; JYH, Japanese Honeysuckle Flower Bud; BLG, Indigowoad Root; QS, Bistort Rhizome; MDP, Tree Peony Root-bark; SDH, Adhesive Rehmannia Root Tuber; CS, Red Paeoniae Trichocarpae; ZC, Redroot Gromwell; CL, Chinese Manyleaf Paris Rhizome; KS, Lightyellow Sophra Root; BXP, Densefruit Pittany Root-bark; TFL, Glabrous Greenbrier Rhizome; GC, Liquorice Root.

From the TCMNPAS database, a total of 203 unique active compounds and 617 corresponding molecular targets were identified across 13 traditional Chinese medicinal herbs after eliminating duplicates. Comprehensive compound and target information is summarized in Supplementary Table S1. The chemical components and predicted targets of XYJDY were imported into Cytoscape software to construct a drug–active compound–target interaction network for XYJDY (Figure 1).

#### 3.1.2 Disease targets associated with psoriasis

**FIGURE 2:**
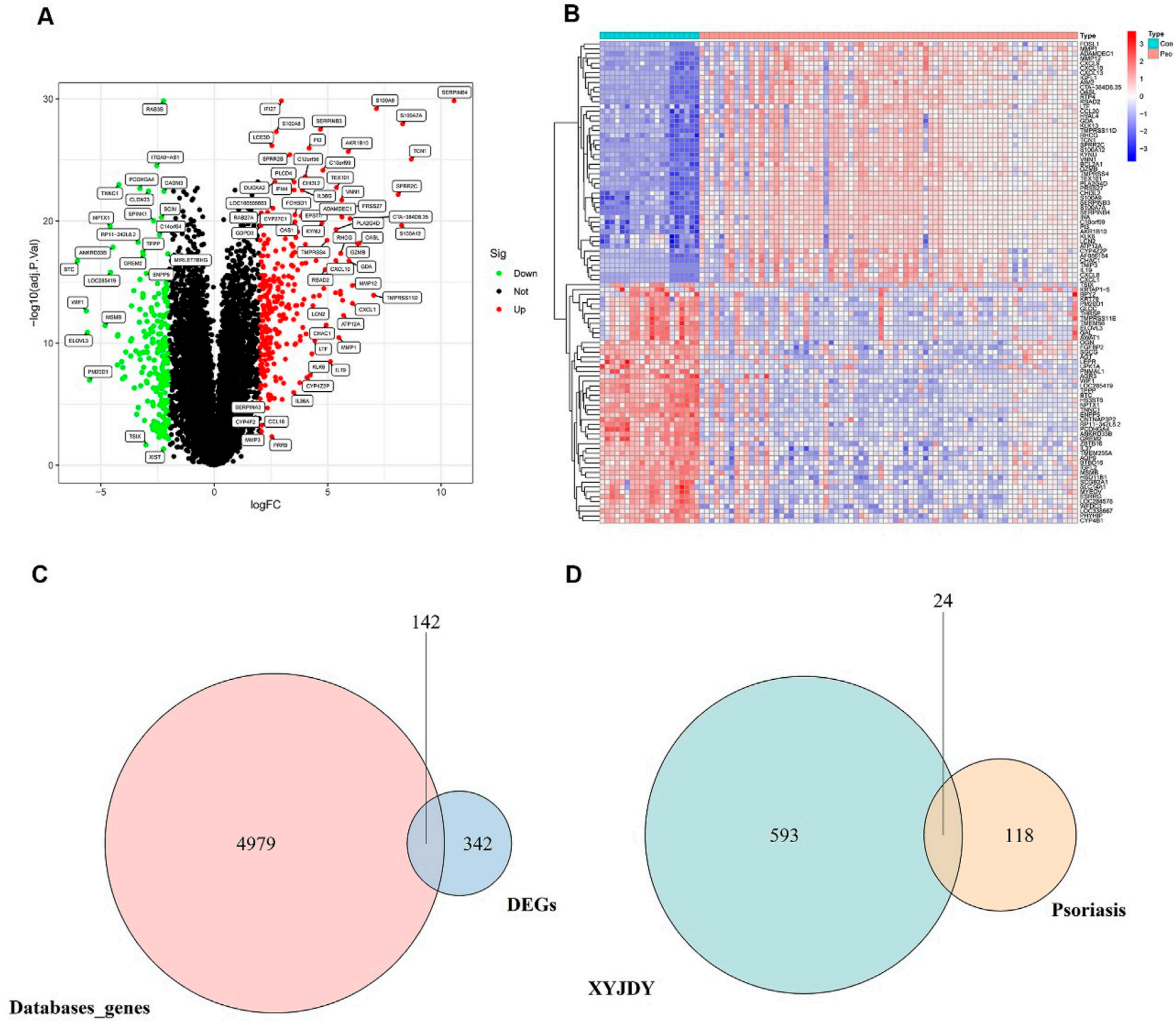
**(A)** Volcano plot of DEGs between psoriasis and healthy control skin samples. **(B)**: Heatmap showing the top 50 most significantly upregulated and downregulated DEGs at the protein-coding level between psoriatic and control skin samples. **(C)**: Venn diagram of the intersection between DEGs from the GEO database and disease-associated genes from public databases. **(D)**: Venn diagram of overlapping targets between XYJDY and psoriasis-related disease targets. Abbreviations: XYJDY, Xiaoxian Jiedu Yin; Con, normal skin tissue; Pso, psoriatic skin tissue; DEGs, differentially expressed genes.

Psoriasis-related DEGs were identified by comparing gene expression profiles between lesional and healthy skin samples. Analysis of three GEO training datasets (GSE78097, GSE182740, and GSE226244) yielded 484 DEGs in total, including 277 upregulated and 207 downregulated genes (Figure 2A). The full list of DEGs is available in Supplementary Table S2. A heatmap was generated to display the top 50 most significantly dysregulated DEGs (Figure 2B).

Furthermore, a total of 5,121 disease-associated targets were compiled from the GeneCards, OMIM, and DisGeNET databases. These were systematically intersected with DEGs identified from the GEO datasets. This integrative analysis ultimately yielded 142 psoriasis-specific disease targets (Figure 2C), the details of which are listed in Supplementary Table S3.

#### 3.1.3 Overlapping targets between XYJDY and psoriasis

Twenty-four overlapping targets were identified by intersecting the predicted targets of XYJDY-derived compounds with the psoriasis-related disease targets (Figure 2D). A detailed list of these overlapping targets is provided in Table 2.

**TABLE 2:** Overlapping targets in XYJDY and psoriasis.

NO.	Uniprot ID	Gene symbol	Protein name
1	O60218	AKR1B10	Aldo-keto reductase family 1 member B10
2	P00491	PNP	Purine nucleoside phosphorylase
3	Q07325	CXCL9	C-X-C motif chemokine 9
4	P02778	CXCL10	C-X-C motif chemokine 10
5	Q16548	BCL2A1	Bcl-2-related protein A1
6	P25025	CXCR2	C-X-C chemokine receptor type 2
7	Q08050	FOXM1	Forkhead box protein M1
8	P78556	CCL20	C-C motif chemokine 20
9	P10145	CXCL8	Interleukin-8
10	O60235	TMPRSS11D	Transmembrane protease serine 11D
11	P48357	LEPR	Leptin receptor
12	O43927	CXCL13	C-X-C motif chemokine 13
13	P09341	CXCL1	Growth-regulated alpha protein
14	P32248	CCR7	C-C chemokine receptor type 7
15	P47989	XDH	Xanthine dehydrogenase/oxidase
16	P03956	MMP1	Interstitial collagenase
17	P06493	CDK1	Cyclin-dependent kinase 1
18	P14780	MMP9	Matrix metalloproteinase-9
19	P21549	AGT	Alanine--glyoxylate aminotransferase
20	P19876	CXCL3	C-X-C motif chemokine 3
21	P01584	IL-1BB	Interleukin-1 beta
22	Q9Y4X3	CCL27	C-C motif chemokine 27
23	P16581	SELE	E-selectin
24	P08254	MMP3	Stromelysin-1

#### 3.1.4 Identification of potential compounds and targets of XYJDY related to psoriasis

**FIGURE 3:**
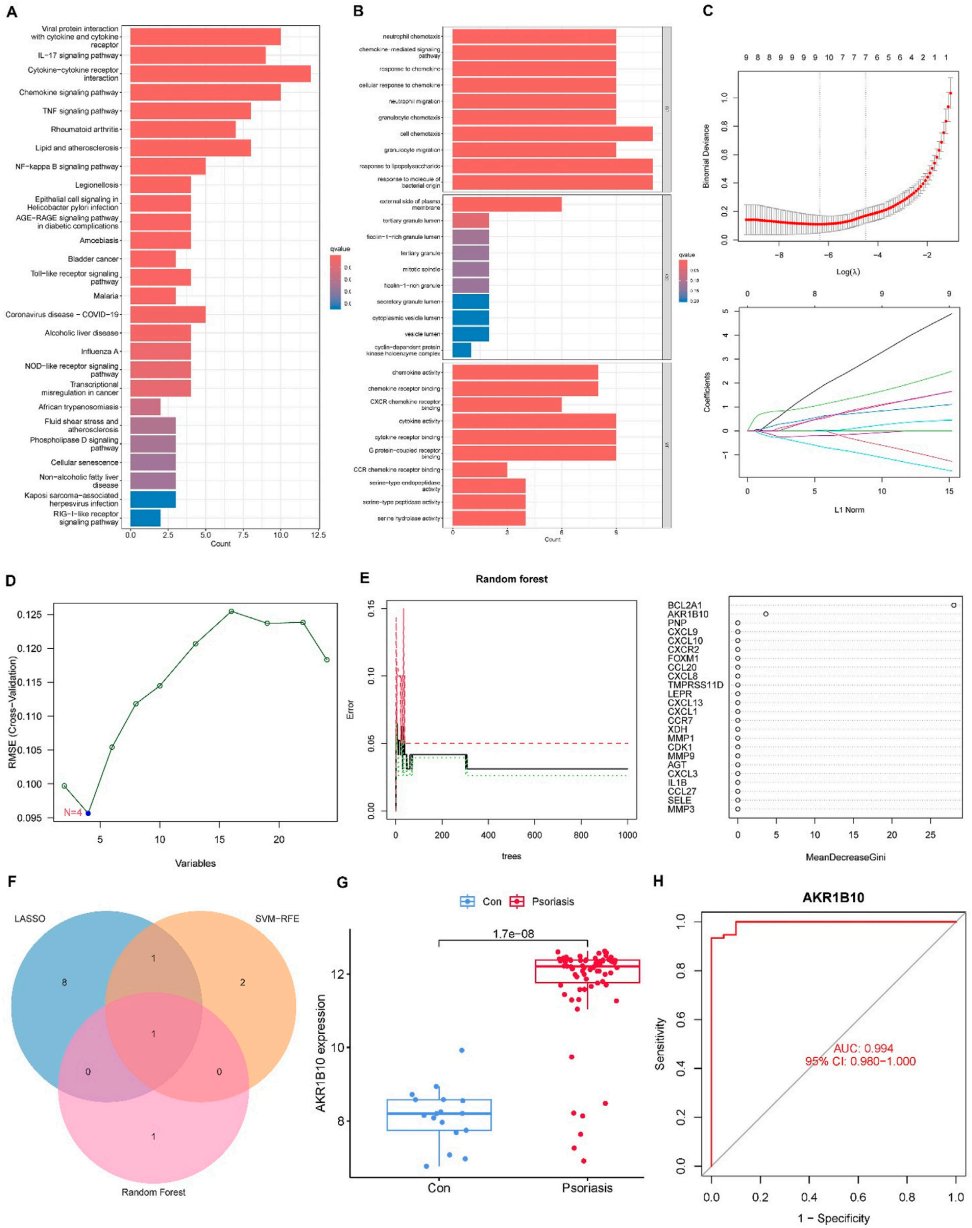
**(A)** KEGG pathway enrichment analysis of the shared targets between XYJDY and psoriasis. **(B)** Gene Ontology enrichment analysis of the shared targets between XYJDY and psoriasis. **(C)** Identification of signature genes using LASSO regression. **(D)** Selection of optimal signature genes via the SVM-RFE algorithm. The x-axis indicates the number of genes, and the y-axis shows the generalization error. A linear trend line illustrates the association between gene count and generalization error. **(E)** Screening of key signature genes using the Random Forest method (left) and ranking of gene importance (right). **(F)** Venn diagram illustrating intersecting genes identified by all three machine learning algorithms. **(G)** Differential expression of AKR1B10 in healthy and psoriatic skin samples. **(H)** ROC curve evaluating the diagnostic accuracy of AKR1B10.

To explore the biological significance of the 24 overlapping targets between XYJDY and psoriasis, GO and KEGG enrichment analyses were performed. KEGG analysis revealed that these targets were predominantly enriched in immune- and inflammation-related pathways, including viral protein interaction with cytokine and cytokine receptors, the IL-17 signaling pathway, the chemokine signaling pathway, cytokine–cytokine receptor interaction, and the TNF signaling pathway (Figure 3A). GO enrichment analysis indicated that in the biological process (BP) category, the targets were mainly involved in cell chemotaxis, response to lipopolysaccharide, and response to molecules of bacterial origin. In the cellular component (CC) category, enrichment was observed in the external side of the plasma membrane and tertiary granule lumen, while in the molecular function (MF) category, the enriched terms included chemokine activity, G protein-coupled receptor binding, and chemokine receptor activity (Figure 3B). These findings suggest that the active components of XYJDY may alleviate psoriasis symptoms by modulating multiple immune and inflammatory pathways.

### 3.2 Machine learning-based identification of key targets and validation using external datasets

To identify potential core targets, LASSO regression was applied to the 24 overlapping genes, resulting in the selection of 10 feature genes: CXCR2, AKR1B10, CCL20, CXCL8, LEPR, CCR7, XDH, MMP9, AGT, and MMP3 (Figure 3C). The SVM-RFE (Support Vector Machine–Recursive Feature Elimination) algorithm identified CXCL8, AKR1B10, CXCL1, and CXCL13 as optimal feature genes (Figure 3D). Random forest analysis further identified BCL2A1 and AKR1B10 as key genes based on their importance scores (Figure 3E). By intersecting the results of the three machine-learning algorithms, AKR1B10 was ultimately selected as the hub gene (Figure 3F). In the validation dataset, AKR1B10 was consistently differentially expressed in psoriasis samples compared to healthy controls, with statistically significant differences observed (Figure 3G). ROC curve analysis showed that AKR1B10 had excellent diagnostic performance, with an AUC of 0.994 (95% CI: 0.980–1.000), indicating high accuracy (Figure 3H). These findings suggest that AKR1B10 may serve as a key therapeutic target of XYJDY in the treatment of psoriasis.

### 3.3 Immune cell infiltration analysis

**FIGURE 4:**
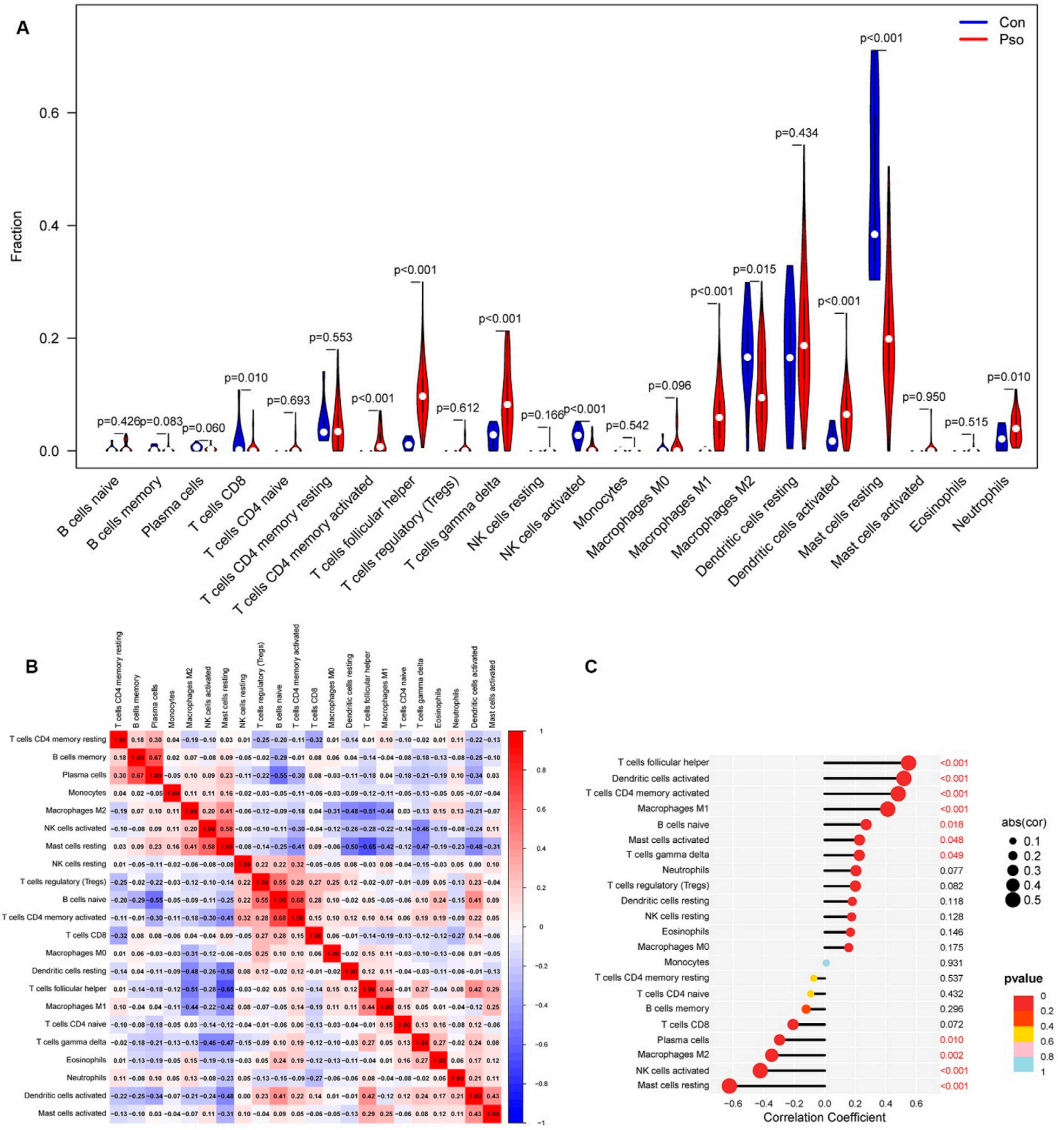
**(A)** Analysis of intergroup differences in immune cells; **(B)**: Immune cell correlation analysis; **(C)**: Correlation between AKR1B10 and immune cell infiltration. con, normal skin tissue; Pso, psoriatic skin tissue.

Immune cell infiltration was analyzed using the CIBERSORT algorithm. After filtering the immune infiltration matrix, a total of 76 reliable samples were obtained, including 65 psoriatic lesion samples and 11 normal control samples. The results showed that activated CD4^+^ memory T cells, follicular helper T (Tfh) cells, γδ T cells, M1 macrophages, activated dendritic cells, and neutrophils were significantly increased in psoriatic lesions. In contrast, CD8^+^ T cells, activated natural killer (NK) cells, and resting mast cells were decreased in psoriatic lesions (Figure 4A). Correlation analysis among immune cell populations revealed several notable associations. Memory B cells were positively correlated with plasma cells (r = 0.67). Resting mast cells were positively correlated with activated NK cells (r = 0.58), while negatively correlated with Tfh cells (r = −0.65) and resting dendritic cells (r = −0.50). Naive B cells showed positive correlations with regulatory T (Treg) cells (r = 0.55) and activated CD4^+^ memory T cells (r = 0.68) but a negative correlation with plasma cells (r = −0.55). Additionally, activated CD4^+^ memory T cells were positively correlated with activated B cells (r = 0.68). These relationships are visualized in Figure 4B, where red indicates a positive correlation, and blue indicates a negative correlation. The expression level of AKR1B10 was positively correlated with activated mast cells, γδ T cells, naive B cells, M1 macrophages, activated CD4^+^ memory T cells, activated dendritic cells, and Tfh cells. Conversely, it was negatively correlated with plasma cells, M2 macrophages, activated NK cells, and resting mast cells (Figure 4C).

### 3.4 scRNA-seq and spatial transcriptomics analysis of AKR1B10

**FIGURE 5:**
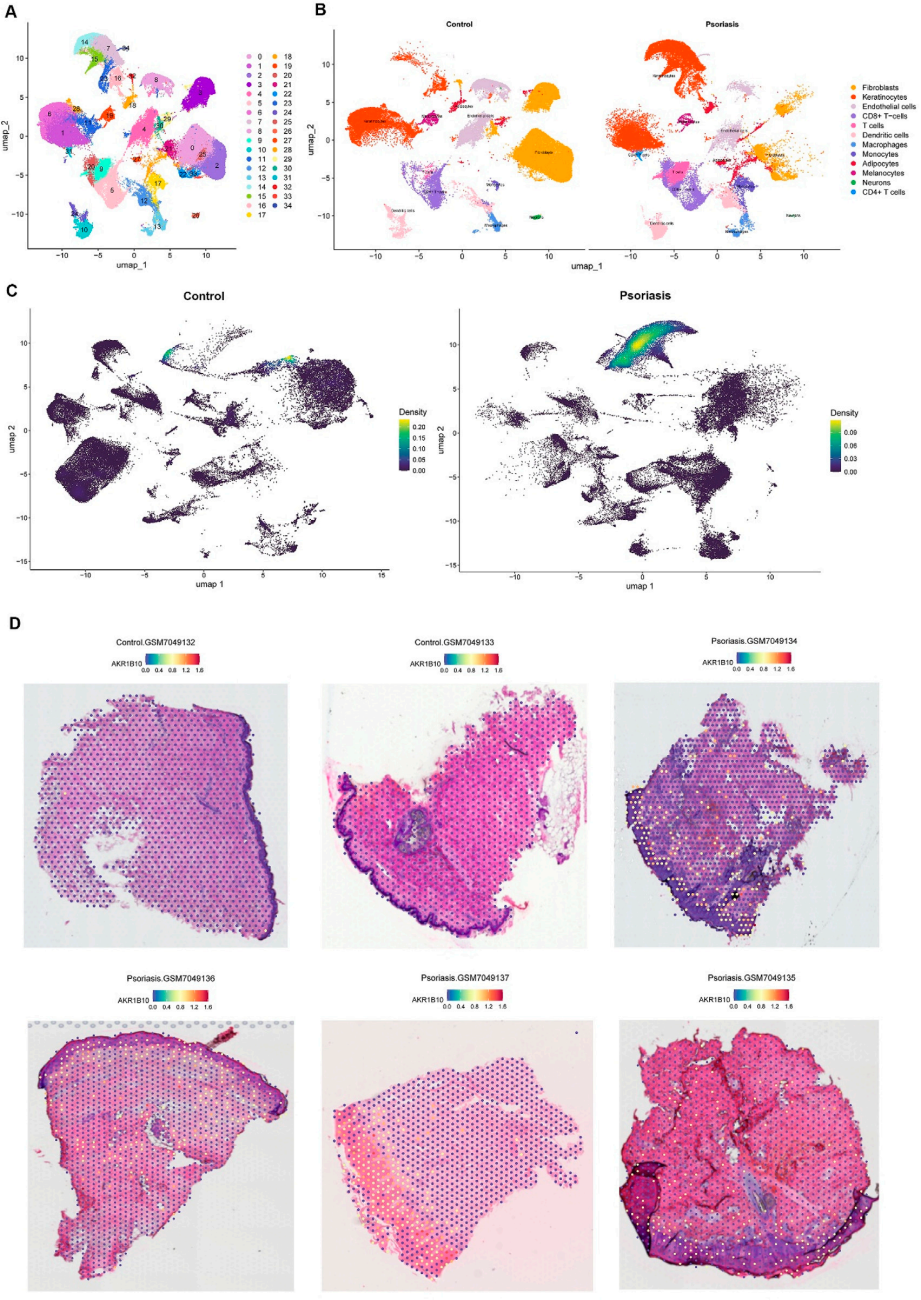
**(A)** UMAP plot showing the clustering of single-cell RNA-sequencing data from all samples. **(B)** UMAP visualization of single-cell clustering in normal and psoriatic skin samples. **(C)** Expression distribution of AKR1B10 in normal and psoriatic skin tissues. **(D)** Spatial localization of AKR1B10 in normal and psoriatic skin tissues, as revealed by spatial transcriptomics.

scRNA-seq data were obtained from the GSE220116 and GSE230842 datasets, comprising a total of 27 samples (13 from psoriatic lesions and 14 from healthy controls). Following data preprocessing procedures—including normalization, standardization, PCA, and UMAP—35 cellular subclusters were identified (Figure 5A). Subsequent cell-type annotation revealed 12 major cell populations: FBs, KCs, ECs, CD8^+^ T cells, CD4^+^ T cells, T cells, DCs, macrophages, monocytes, adipocytes, melanocytes, and neurons (Figure 5B). In the psoriatic group, AKR1B10 expression was predominantly enriched in KCs, with minimal expression observed in fibroblasts, melanocytes, adipocytes, and other clusters (Figure 5C). ST analysis further demonstrated that AKR1B10 expression was almost absent in the control group but was markedly elevated in the psoriatic group, primarily localized within the epidermal layer (Figure 5D).

### 3.5 XYJDY-medicated serum inhibits AKR1B10 expression in IL-17A-induced HaCaT cells

As shown in Supplementary Figure S1A, no significant changes in HaCaT cell proliferation were observed across varying IL-17A concentrations after 24 h of exposure. However, after 48 h of stimulation, treatment with 200 ng/mL IL-17A resulted in a markedly increased proliferation rate compared to the untreated control group (P < 0.01; Supplementary Figure S1B). Based on these findings, 200 ng/mL IL-17A stimulation for 48 h was established as the optimal induction condition for HaCaT cells in this study.

To evaluate the therapeutic effect of XYJDY on the psoriasiform inflammatory model of HaCaT cells, cells were stimulated with IL-17A (200 ng/mL for 48 h) and subsequently treated with either blank control serum or XYJDY-medicated serum. As shown in Supplementary Figure S2A,B, treatment with blank control serum at all tested concentrations significantly enhanced HaCaT cell proliferation at both 24 h and 48 h when compared to the normal group (*P* < 0.001). However, no significant differences were observed among the 5%, 10%, 15%, and 20% blank serum groups relative to the IL-17A-treated model group. In contrast, after 24 h of intervention, treatment with 15% and 20% XYJDY-medicated serum significantly reduced HaCaT cell proliferation compared to the model group (*P* < 0.001; Supplementary Figure S2C). After 48 h, a significant reduction in proliferation was observed in the 5%, 10%, and 15% XYJDY-medicated serum groups (*P* < 0.001; Supplementary Figure S2D). Therefore, 15% XYJDY-containing serum was used in this study to intervene in HaCaT cells for 24 h as the optimal administration concentration and time for subsequent experiments.

**FIGURE 6:**
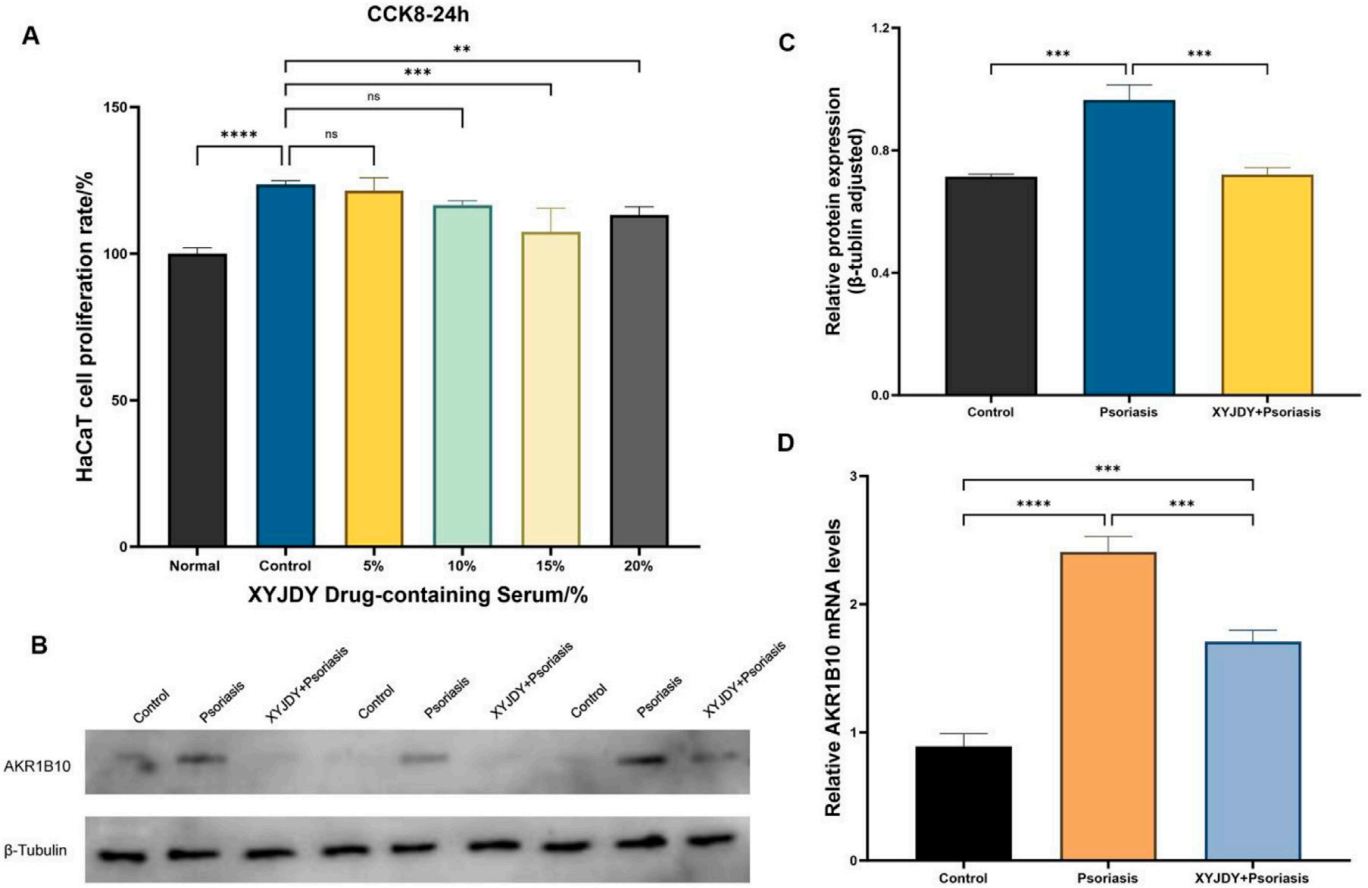
**(A)** CCK-8 Detection of cell proliferation rate in a psoriasis-like inflammation model of HaCaT cells after 24 24h of intervention with XYJDY drug-containing serum; **(B)** Western blot analysis of AKR1B10 expression in HaCaT cells induced by IL-17A in different treatments; **(C)** Western blot detection of AKR1B10 expression in each group (n = 3/group); **(D)** RT-qPCR analysis to detect the expression of AKR1B10 mRNAs in each group (n = 3/group); **P < 0.01, ***P < 0.001, ****P < 0.0001.

As predicted, WB and RT-qPCR analyses revealed significantly lower AKR1B10 expression in IL-17A-induced HaCaT cells following 24 h treatment with XYJDY-medicated serum compared to the model group (Figures 6B,C). This suggests that XYJDY-medicated serum may exert its anti-psoriasis effect through inhibition of AKR1B10.

## 4 Discussion

Although the pathophysiological mechanisms of psoriasis remain incompletely understood, current evidence highlights key pathological features, including hyperproliferation of KCs, immune cell infiltration, dysregulated proinflammatory cytokine networks, and aberrant dermal angiogenesis (Gao et al., 2025). Given the disease’s multifaceted and multitargeted nature, the pursuit of novel treatment strategies with both robust efficacy and favorable safety profiles remains a critical priority in dermatology. TCM, in contrast to conventional therapies, offers distinctive advantages in treating psoriasis, including syndrome differentiation, individualized diagnostic approaches, and therapeutic flexibility. Backed by centuries of clinical practice, TCM’s diverse treatment principles, herbal formulas, and medicinal components have consistently demonstrated reliable efficacy and safety, underscoring their potential clinical value and research relevance (Su et al., 2021).

To clarify the core molecular mechanisms underlying XYJDY, a traditional Chinese medicine formula, in the treatment of psoriasis, we employed a variety of strategies. First, we integrated bioinformatics and network pharmacology approaches to predict the potential targets of XYJDY in treating psoriasis. Following this, we applied several machine learning algorithms to systematically cross-screen the predicted targets, ultimately identifying AKR1B10 as a key candidate target mediating the pharmacological activity of XYJDY. This data-driven approach significantly narrowed down the scope of potential targets, providing a clear direction for subsequent research. We then validated the expression pattern of AKR1B10 in psoriasis and its association with the disease using immune cell infiltration analysis, scRNA-seq, and ST. Finally, *in vitro* experiments were conducted using XYJDY-containing serum to intervene in the IL-17A-induced HaCaT cell psoriasis-like inflammation model, confirming the impact of XYJDY on AKR1B10 expression.

GO and KEGG enrichment analyses of XYJDY-related targets suggest that its core therapeutic mechanisms in psoriasis may involve the regulation of key pathogenic pathways and biological processes. Specifically, KEGG analysis demonstrated significant enrichment in the IL-17 signaling pathway, TNF signaling pathway, and cytokine-cytokine receptor interaction pathway. Complementary GO analysis revealed that XYJDY targets are notably enriched in biological processes such as cell chemotaxis, chemokine activity, G protein-coupled receptor (GPCR) binding, and chemokine receptor activity. Overall, these findings underscore the multifaceted therapeutic potential of XYJDY, indicating that its efficacy is achieved through the coordinated regulation of multiple signaling pathways and molecular targets.

Importantly, although AKR1B10 has been previously recognized as a key regulatory molecule in the onset and progression of inflammatory diseases (Guo et al., 2024; Sumantran et al., 2016), including psoriasis, our study provides substantial new evidence that establishes it as a core functional target of XYJDY. AKR1B10 is involved in NF-κB, MAPK signaling, and retinoid metabolism, driving the expression of pro-inflammatory cytokines such as IL-6 and IL-1β. Additionally, AKR1B10 contributes to the biosynthesis of long-chain fatty acids and the regulation of lipid metabolism, highlighting its potential central role in mediating the interaction between inflammation and metabolic processes (Cheng et al., 2018; Endo et al., 2021). The expression of AKR1B10 is regulated by various mechanisms, including KEAP1 mutations, EGF exposure, and IRAK1 overexpression. *In vitro* experiments have demonstrated that silencing AKR1B10 effectively suppresses excessive proliferation and migration of HaCaT cells (Gao et al., 2017), further highlighting the potential therapeutic value of this target in psoriasis treatment.

Our integrated immune infiltration analysis further revealed that the expression of AKR1B10 in psoriatic lesions is significantly positively correlated with pro-inflammatory Th17-associated immune cells (including activated CD4^+^ memory T cells and Tfh cells), γδ T cells, and M1 macrophages, while negatively correlated with immune-regulatory M2 macrophages and NK cells. This strongly suggests that AKR1B10 promotes a pro-inflammatory immune microenvironment that sustains psoriasis inflammation. Furthermore, scRNA-seq and ST analyses offered novel insights at both spatial and cellular resolutions: compared to normal skin, AKR1B10 expression is significantly upregulated in psoriatic lesions. scRNA-seq precisely localized its major enrichment to keratinocytes (KCs), while ST revealed its expression extending from the epidermis into the dermis. This spatial expression pattern indicates a potential role for AKR1B10 in mediating immune crosstalk between different skin layers. To determine whether AKR1B10 acts as a target of XYJDY, we established an IL-17A-induced HaCaT inflammatory model. Treatment with XYJDY-containing serum resulted in a significant reduction in AKR1B10 expression. The observed downregulation of AKR1B10 provides crucial experimental support for our computational predictions and pharmacological hypotheses, suggesting that XYJDY can effectively regulate the expression of this critical target in a psoriasis-like inflammation model.

In summary, this study, which integrates network pharmacology, computational predictions, multi-omics validation, and *in vitro* experiments, provides evidence supporting AKR1B10 as a potential key target mediating the anti-psoriatic effects of XYJDY. Furthermore, *in vitro* experiments using the IL-17A-induced HaCaT inflammatory model demonstrated that XYJDY-containing serum significantly reduced AKR1B10 expression. As a molecule confirmed to drive pro-inflammatory signaling and lipid metabolism dysregulation in psoriatic lesions, the downregulation of AKR1B10 offers a plausible mechanistic link to explain the therapeutic activity of XYJDY. Based on our findings, we propose a hypothesis: XYJDY may (at least partially) exert its beneficial effects by targeting AKR1B10, thereby potentially inhibiting its downstream pro-inflammatory and metabolic pathways. This targeted mechanism may contribute to the restoration of keratinocyte homeostasis and the regulation of the dysregulated immune microenvironment, providing molecular insights into multi-pathway synergistic interventions for psoriasis.

Although the preliminary findings presented in this study provide a foundation for future research, several limitations must be acknowledged. First, some of the analytical data were sourced from public databases, which may be impacted by incomplete annotations and inconsistent quality control. Additionally, the variability in literature sources introduces a risk of systematic bias. To mitigate this, we utilized high-resolution single-cell and spatial transcriptomics validation (scRNA-seq and ST), achieving cell-type-specific and spatial cross-validation of the expression patterns of key targets, which strengthened the robustness of our conclusions. Second, while *in vitro* evidence demonstrates that XYJDY-containing serum significantly inhibits AKR1B10 expression in HaCaT cells, its downstream signaling networks have not been fully mechanistically validated. Future research will focus on this aspect to comprehensively evaluate its pharmacodynamic effects and target involvement. Lastly, considering that the pharmacological effects of traditional Chinese medicine may partially rely on *in vivo* metabolites, future studies could use LC-MS/MS and spatial metabolomics to explore the metabolic transformation and tissue-specific distribution of XYJDY-containing serum. Such analyses may further enrich our understanding of its pharmacological basis in psoriasis.

## 5 Conclusion

In conclusion, this study demonstrates that XYJDY exerts anti-psoriatic effects through multi-target and multi-pathway mechanisms, with AKR1B10 emerging as a potential core target. Our findings provide a scientific foundation for clinical translation and mechanistic research of this TCM formula. Further investigation into AKR1B10-dependent signaling networks is warranted to validate the proposed mechanisms and their pathophysiological relevance.

## Data Availability

The datasets analysis for this study can be found in the GEO public database (https://www.ncbi.nlm.nih.gov/geo/).
