# Correction: Investigation of the mechanism of Xiaoyin Jiedu Yin in the treatment of psoriasis based on bioinformatics, machine learning

**DOI:** 10.3389/fchem.2025.1685169

**Published:** 2025-09-17

**Authors:** Ru-Nan Fang, Yang Zhou, Yang Shen, Yuan Sun, Jian-Hong Li

**Affiliations:** 1 Department of Dermatology, Dongzhimen Hospital, Beijing University of Chinese Medicine, Beijing, China; 2 Department of Endocrinology, Guang’anmen Hospital, Beijing University of Chinese Medicine, Beijing, China

**Keywords:** psoriasis, bioinformatics, network pharmacology, Xiaoyin Jiedu Yin, machine learning, single-cell RNA sequencing

There was a mistake in Figure 6B as published. The β-Tubulin bands were indentified as target protein, and the AKR1B10 bands were identified as internal control. The β-Tubulin bands should serve as the internal control, while the AKR1B10 bands represent the target protein. The corrected figure appears below.

**FIGURE 6:**
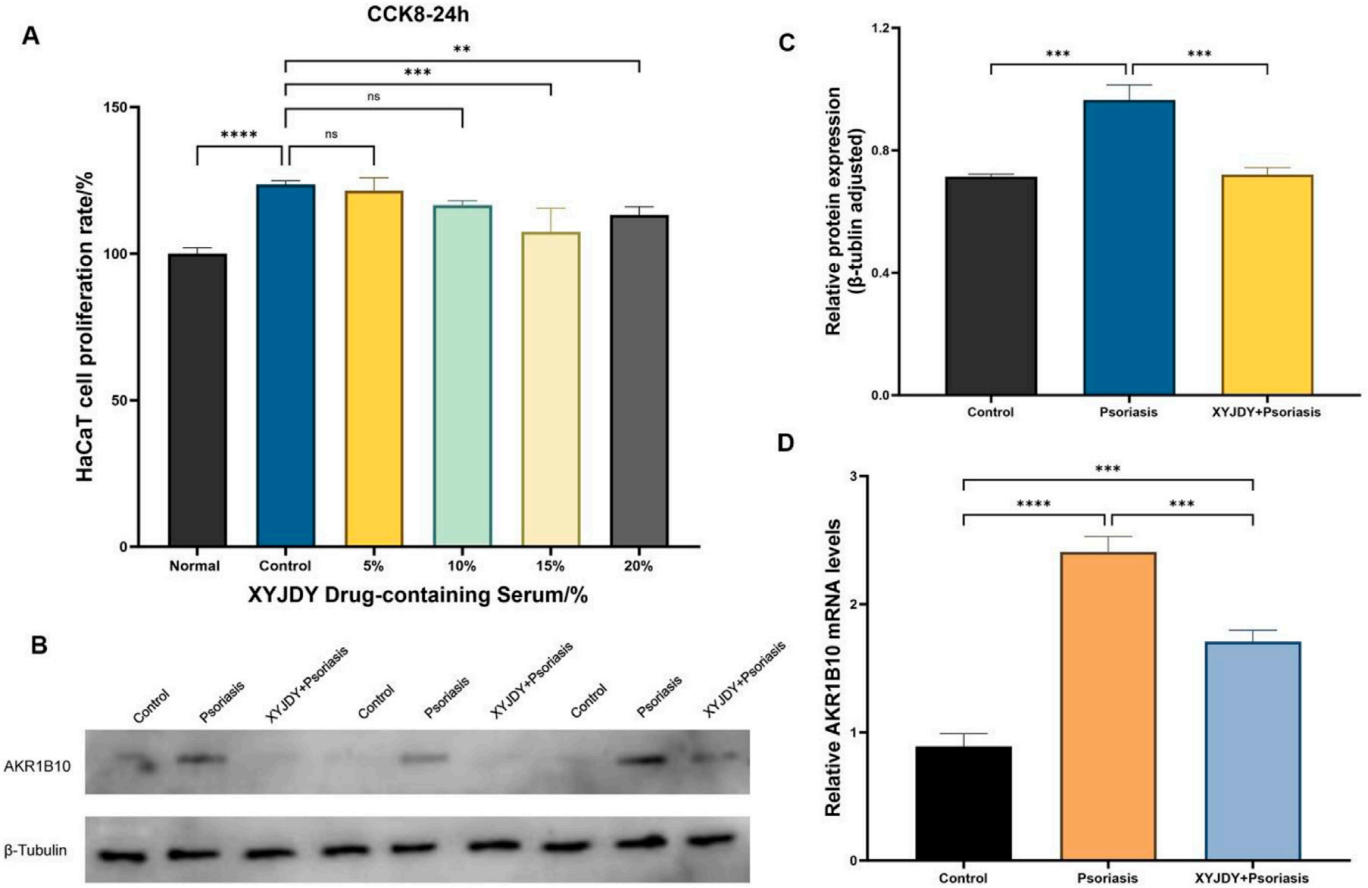
**(A)** CCK-8 Detection of cell proliferation rate in a psoriasis-like inflammation model of HaCaT cells after 24 24 h of intervention with XYJDY drug-containing serum; **(B)** Western blot analysis of AKR1B10 expression in HaCaT cells induced by IL-17A in different treatments; **(C)** Western blot detection of AKR1B10 expression in each group (n = 3/group); **(D)** RT-qPCR analysis to detect the expression of AKR1B10 mRNAs in each group (n = 3/group); **P < 0.01, ***P < 0.001, ****P < 0.0001.

The original article has been updated

