# Supplementary material for: Investigation of the mechanism of Xiaoyin Jiedu Yin in the treatment of psoriasis based on bioinformatics, machine learning
Source: Front Chem. 2025 Jul 15;13:1623449. doi: 10.3389/fchem.2025.1623449 (PMC12303907; doi:10.3389/fchem.2025.1623449)
Supplement: Supplementary file 2 [file DataSheet1.docx]

**A B**


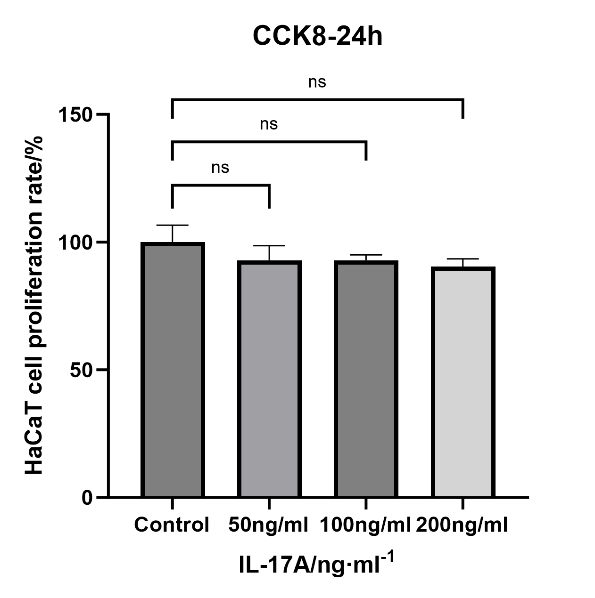

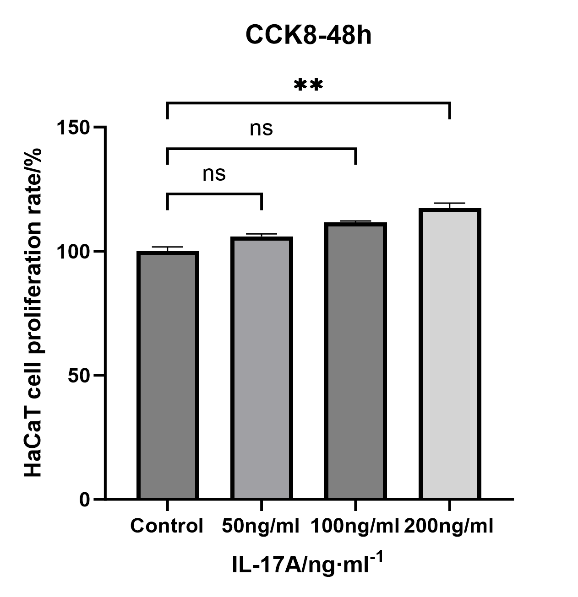


Supplementary Figure1 A. CCK-8 Detection of cell proliferation rate in HaCaT cells 24h after IL-17A induction; B. CCK-8 Detection of cell proliferation rate in HaCaT cells 48h after IL-17A induction. ***P* < 0.01.

**A B**


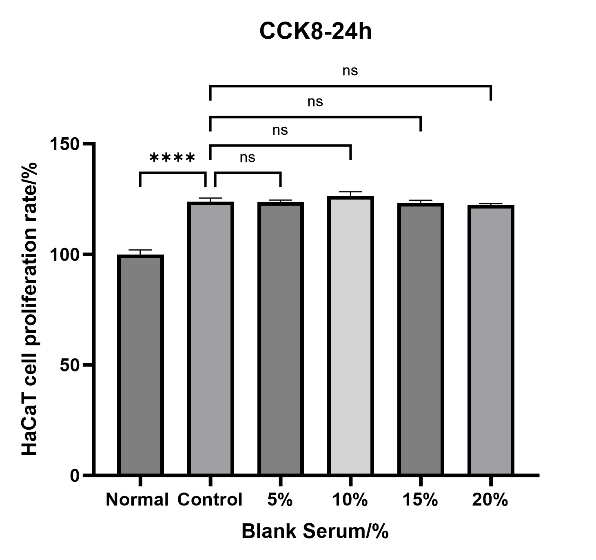

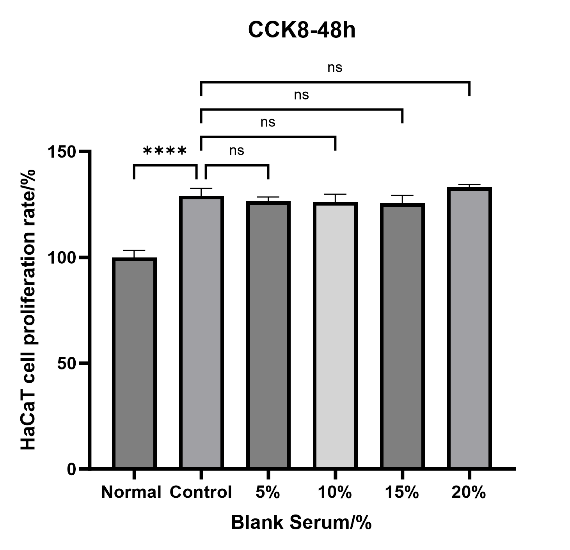


**C**


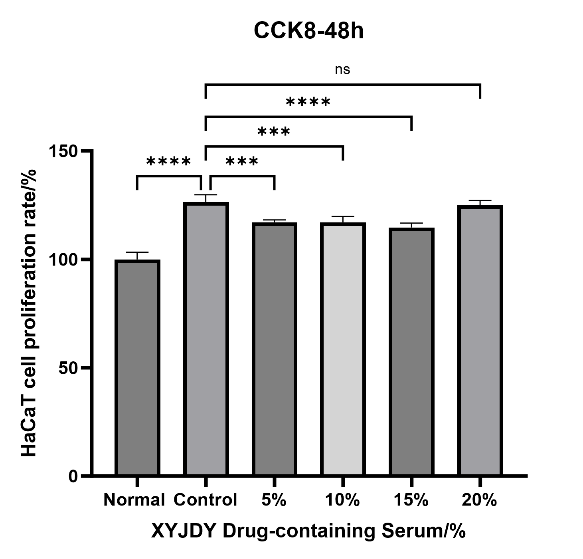


Supplementary Figure2 A. CCK-8 Detection of cell proliferation rate in a psoriasis-like inflammation model of HaCaT cells after 24 h of blank serum intervention; B.CCK-8 Detection of cell proliferation rate in a psoriasis-like inflammation model of HaCaT cells after 48 h of blank serum intervention; C. CCK-8 Detection of cell proliferation rate in a psoriasis-like inflammation model of HaCaT cells after 48h of intervention with XYJDY drug-containing serum. ***P* < 0.01, ****P* < 0.001, *****P*< 0.0001.
